# SHEA position statement on pandemic preparedness for policymakers: building a strong and resilient healthcare workforce

**DOI:** 10.1017/ice.2024.62

**Published:** 2024-07

**Authors:** David B. Banach, Trini A. Mathew, Lynne Jones Batshon, Westyn Branch-Elliman, Ghinwa Dumyati, Sarah Haessler, Vincent P. Hsu, Robin L. P. Jump, Anurag N. Malani, Rekha K. Murthy, Steven A. Pergam, Erica S. Shenoy, David J. Weber

**Affiliations:** 1 School of Medicine, University of Connecticut, Farmington, CT, USA; 2 Yale School of Public Health, New Haven, CT, USA; 3 HealthTAMCycle3, PLLC, Troy, MI, USA; 4 Corewell Health, Taylor, MI, USA; 5 School of Medicine, Wayne State University, Detroit, and Oakland University William Beaumont, Rochester, MI, USA; 6 Society for Healthcare Epidemiology of America (SHEA), Arlington, VA, USA; 7 Veterans Affairs Boston Healthcare System, Boston, MA, USA; 8 Harvard Medical School, Boston, MA, USA; 9 University of Rochester Medical Center, Rochester, NY, USA; 10 Center for Community Health, Rochester, NY, USA; 11 Baystate Medical Center, Springfield, MA, USA; 12 University of Massachusetts Chan Medical School – Baystate, Springfield, MA, USA; 13 AdventHealth, Altamonte Springs, FL, USA; 14 School of Medicine, Loma Linda University, Loma Linda, CA, USA; 15 Geriatric Research Education and Clinical Center (GRECC) at the Veterans Affairs Pittsburgh Healthcare System, Pittsburgh, PA, USA; 16 University of Pittsburgh School of Medicine, Pittsburgh, PA, USA; 17 Trinity Health Michigan, Ann Arbor, MI, USA; 18 Cedars-Sinai, Los Angeles, CA, USA; 19 David Geffen School of Medicine at UCLA, Los Angeles, CA, USA; 20 Fred Hutchinson Cancer Research Center, Seattle, WA, USA; 21 University of Washington, Seattle, WA, USA; 22 Seattle Cancer Care Alliance, Seattle, WA, USA; 23 Massachusetts General Hospital, Boston, MA, USA; 24 Harvard Medical School, Boston, MA, USA; 25 Mass General Brigham, Boston, MA, USA; 26 University of North Carolina, Chapel Hill, NC, USA

## Abstract

Throughout the COVID-19 pandemic, many areas in the United States experienced healthcare personnel (HCP) shortages tied to a variety of factors. Infection prevention programs, in particular, faced increasing workload demands with little opportunity to delegate tasks to others without specific infectious diseases or infection control expertise. Shortages of clinicians providing inpatient care to critically ill patients during the early phase of the pandemic were multifactorial, largely attributed to increasing demands on hospitals to provide care to patients hospitalized with COVID-19 and furloughs.^1^ HCP shortages and challenges during later surges, including the Omicron variant-associated surges, were largely attributed to HCP infections and associated work restrictions during isolation periods and the need to care for family members, particularly children, with COVID-19. Additionally, the detrimental physical and mental health impact of COVID-19 on HCP has led to attrition, which further exacerbates shortages.^2^ Demands increased in post-acute and long-term care (PALTC) settings, which already faced critical staffing challenges difficulty with recruitment, and high rates of turnover. Although individual healthcare organizations and state and federal governments have taken actions to mitigate recurring shortages, additional work and innovation are needed to develop longer-term solutions to improve healthcare workforce resiliency. The critical role of those with specialized training in infection prevention, including healthcare epidemiologists, was well-demonstrated in pandemic preparedness and response. The COVID-19 pandemic underscored the need to support growth in these fields.^3^ This commentary outlines the need to develop the US healthcare workforce in preparation for future pandemics.

## Background

Throughout the COVID-19 pandemic, many areas in the United States experienced healthcare personnel (HCP) shortages tied to a variety of factors. Infection prevention programs, in particular, faced increasing workload demands with little opportunity to delegate tasks to others without specific infectious diseases or infection control expertise. Shortages of clinicians providing inpatient care to critically ill patients during the early phase of the pandemic were multifactorial, largely attributed to increasing demands on hospitals to provide care to patients hospitalized with COVID-19 and furloughs.^1^ HCP shortages and challenges during later surges, including the Omicron variant-associated surges, were largely attributed to HCP infections and associated work restrictions during isolation periods and the need to care for family members, particularly children, with COVID-19. Additionally, the detrimental physical and mental health impact of COVID-19 on HCP has led to attrition, which further exacerbates shortages.^2^ Demands increased in post-acute and long-term care (PALTC) settings, which already faced critical staffing challenges difficulty with recruitment, and high rates of turnover. Although individual healthcare organizations and state and federal governments have taken actions to mitigate recurring shortages, additional work and innovation are needed to develop longer-term solutions to improve healthcare workforce resiliency. The critical role of those with specialized training in infection prevention, including healthcare epidemiologists, was well-demonstrated in pandemic preparedness and response. The COVID-19 pandemic underscored the need to support growth in these fields.^3^ This commentary outlines the need to develop the US healthcare workforce in preparation for future pandemics.

## Rationale

### Healthcare workforce needed for the future

The COVID-19 pandemic laid bare the weak spots and vulnerabilities of healthcare delivery and exposed the lack of a robust and nimble healthcare workforce across the continuum of care, creating substantial challenges in responding to an infectious disease crisis. In preparation for future pandemics, the healthcare workforce will need to have an agile knowledge and skill set. Experience in the COVID-19 response has demonstrated the expansion of remote healthcare delivery, including telehealth, in the diagnosis and treatment of pandemic agents and continuing routine medical care in a manner supporting safety of HCP and patients.^4^

Technological innovations can be used to improve planning and allocation of limited HCP and other resources. With investment, artificial intelligence and machine learning technologies can be used to detect infectious disease threats. The early identification of threats through technological advances can be leveraged alongside the human workforce, including HCP, to accelerate and support pandemic preparedness and response. Examples include the United States Centers for Disease Control and Prevention Center for Forecasting and Outbreak Analytics and public-private partnerships that utilize data sharing and analytics to predict demands on the healthcare workforce. Prediction modeling could be coupled with reduction in administrative burden, for example for credentialling, to allow the healthcare workforce to be rapidly deployed if needed in an emergency situation. Furthermore, one unified federal licensing system can be adopted during emergencies, to allow for HCP to be credentialed across multiple state lines if technical expertise or frontline support is needed. This approach can reduce the time needed for an effective response to meet the needs of patients.

Identification of early signals of potential pandemics, including responses to any changing landscape of a myriad of global pathogens and environmental interactions, requires a change in our existing paradigm and definition of healthcare workforce. Currently, the healthcare workforce in acute care and other healthcare settings, including PALTC, is composed of key categories with job descriptions across the continuum of care (Table 1). To prepare for future pandemics that may be associated with emerging pathogens with pandemic potential, the response will require healthcare facilities to expand the pool of HCP, including those involved with direct clinical care and nonclinical support staff. This can involve dynamic strategies that increase the overall number of available HCP in specific settings to meet demands and cross-train existing HCP. Facilities will also need robust expertise in data analytics; business processes; technology and robotic support; climate scientists and environmental microbiologists with knowledge and ability to develop predictive models, respond to external threats, and work collaboratively with clinicians and policy makers.

**Table 1.** Examples of members in the healthcare workforce impacted by pandemic preparedness and response*

	Acute Care	Post-Acute and Long-Term Care	Ambulatory, including dental	Other (dialysis, infusion centers, same-day surgery)
**HCP who engage in direct patient care**	Nurses, nurse aides, nurse practitioners, physicians, physician assistants, respiratory therapists	Nurses, nurse aides, nurse practitioners, physicians, physician assistants, respiratory therapists	Dentists, nurses, nurse aides, nurse practitioners, physicians, physician assistants	Nurses, nurse aides, nurse practitioners, physicians, physician assistants
**HCP not providing direct patient care who have direct contact with the patient or their environment** **	Clergy, dietary, environmental management services, phlebotomy, physical therapy/occupational therapy, radiology technicians, transportation and escort personnel, volunteers	Clergy, dietary, environmental management services, phlebotomy, physical therapy/occupational therapy, radiology technicians, transportation and escort personnel, volunteers	Phlebotomy, physical therapy/occupational therapy, radiology technicians, transportation and escort personnel, volunteers	Dialysis technicians, phlebotomy, transportation and escort personnel, volunteers
**HCP not providing direct patient care who have indirect contact with the patient or their environment**	Administrative, billing, facilities and maintenance, laundry, pharmacists	Administrative, billing, facilities and maintenance, laundry, pharmacists	Administrative, billing, environmental management services, facilities and maintenance	Administrative, billing, environmental management services, facilities and maintenance, laundry

* This list is not intended to be all-inclusive of all members of the healthcare workforce. Additional stakeholders in outbreak response and incident management can be referenced in the SHEA Outbreak Response and Training Program accessible at Potential Stakeholders—Outbreak Response Tool Kits Outbreak Response Tool Kits (guidelinecentral.com)

** includes body substances (e.g., blood, tissue, and specific body fluids); contaminated medical supplies, devices, and equipment; contaminated environmental surfaces; or contaminated air

**Table 2.** Building a strong and resilient healthcare workforce: challenges, recommendations to policymakers, and examples

Challenge	Recommendation (*examples*)
The healthcare workforce is highly specialized with limited capacity to adapt to changing healthcare needs in a pandemic to provide care to a surge of patients with infections while supporting ongoing care of all patients	Expand the functionality of the existing healthcare workforce, with cross-training of healthcare personnel to adapt to changing needs during a pandemic *Support necessary educational programs to train staff in emergency response and care provision that would be needed in an infectious disease pandemic or crisis*
Many members of the healthcare workforce are inadequately trained in fundamental infection control practices	Provide tailored education to frontline HCP on infection prevention and specific roles and responsibilities during infectious diseases outbreaks with routine ongoing evaluation of competency *Support programs led by experts in healthcare epidemiology and infection control (SHEA, APIC, state and federal public health agencies) to provide pathogen-specific and core infection prevention practices* *Support research into best practices and strategies for sustaining knowledge obtained during trainings* *Create standardized assessments of competency for core infection prevention interventions (e.g., hand hygiene, correct selection, donning, and doffing of PPE)*
In the setting of a pandemic there may be barriers to the provision of care using standard, generally in-person, care delivery modalities	Support research on best practices for the implementation and assessment of telemedicine and the expansion of the workforce. Create an evidence-informed framework for providing telemedicine both for diagnosis and treatment of potential pandemic agents and maintaining routine patient care during a pandemic or crisis. *Evaluate strategies of healthcare provision using different modalities, such as telemedicine, that may be deployed in a pandemic and their short and long-term impact on patient care* *Evaluate the sustainability of telemedicine as a modality for providing care* *Evaluate impacts of short-term and long-term telemedicine programs on a variety of clinical outcomes* *Explore the use of AI and robotics in supporting care that may reduce the demand on human resources*
During a pandemic, patient care needs and staffing shortages are dynamic and variable	Support collaborations across scientific communities and health systems, to better understand and respond to threats to the healthcare workforce and infrastructure resilience in various settings *Elimination of administrative barriers to workforce mobility/deployment to areas of need, standardized emergency credentialing processes, international oversight and coordination of workforce supply* *Create a national system for credentialling healthcare personnel and eliminate inter-state barriers to practice and movement* *Develop models of current and future staffing needs to assist with deployment of human resources*
The clinical workforce is segregated from the public health workforce and infrastructure	Strengthen relationships and collaboration between communities, state and local public health departments, and the healthcare settings they serve. Support expansion of the local healthcare workforce to assist with pandemic preparedness and response activities, including strengthening ties with public healthcare personnel. These partnerships should include diversity, equity, and inclusion in the development of the healthcare workforce. *Routine communication between public health departments and clinical leadership, recruitment and support of diversity in the clinical and public health workforce*
Expertise in healthcare epidemiology and infection prevention in acute, post-acute, and other healthcare settings is limited	Recognize the expertise of healthcare epidemiologists and infection preventionists and provide dedicated time and resources to support their activities and roles in pandemic preparedness and response across healthcare facilities, including expansion of capacity, when needed *Allocate resources to support time needed to dedicate in pandemic response, develop strategies to strengthen the infection prevention workforce during an infectious disease pandemic* *Develop reimbursement frameworks for supporting infection prevention and healthcare epidemiology expertise*

### Healthcare workforce shortages

A robust, well-trained, and dynamic healthcare and public health workforce is critical in adequately preparing for and addressing the clinical and public health needs of the population. Prior to the COVID-19 pandemic, there were already identified national shortages of physicians and nurses as well as public health professionals.^5^

### Changes in the healthcare workforce may exacerbate shortages and healthcare disparities

In addition to the existing shortages, the ageing healthcare workforce may result in further challenges in meeting demands, particularly in underserved areas. A substantial proportion of the healthcare workforce, including physicians and nurses are 55 years of age or older.^6,7^ In addition to higher risk of severe infection from COVID-19, older HCP are closer to retirement and are less likely to remain working full-time, potentially exacerbating current challenges if a pipeline of future HCP with relevant expertise is not supported. Substantial geographical variability within the ageing healthcare workforce intensifies existing HCP shortages in underserved communities.

### The direct impact of COVID-19 on the healthcare workforce

In the early months of the COVID-19 pandemic, there was a notable decline in the healthcare workforce across healthcare sectors, generally attributed to job loss, reductions in elective clinical services, and attrition.^8^ The pandemic also had an appreciable adverse impact on the mental and physical health of HCP^9,10^ resulting in further attrition of the healthcare workforce and exacerbating existing shortages and disparities. In addition to those who have already left healthcare, a large proportion of HCP have described an intention to leave their jobs in the near future, potentially exacerbating current challenges and creating vulnerabilities if another emergency occurs in the near future.^11^ Public health workers have seen similarly high rates of mental health challenges during the COVID-19 pandemic.^12^

The COVID-19 pandemic demonstrated the importance of a dynamic healthcare workforce, one that can adapt to the changing needs of the population. In the early phase of the COVID-19 pandemic, there was an immediate and intense need to mobilize the healthcare workforce to provide care to large numbers of patients who were acutely ill, many of whom required hospitalization and intensive care. After the initial wave of infections, HCPs were needed to provide care to patients who had delayed care during the initial pandemic surge, many of whom had delayed time-sensitive care, including elective surgeries and interrupted care for chronic medical conditions.^13^ Additionally, the pandemic created new needs following the initial surge of infections including infection prevention-related care, such as rapid deployment of vaccines and prophylactic medications and long-term care for those recovering from the first wave. The ability of the HCP to sustain resiliency and pivot through various phases of a pandemic while maintaining focus on infection prevention and the physical and mental health of the workforce will be critical in future pandemic preparedness.

The COVID-19 pandemic also highlighted the geographical maldistribution of HCP in the United States.^1^ As the pandemic evolved regionally, many areas of the country with existing HCP shortages were disproportionately impacted by infection spread. This coupling of high community transmission and inadequate staffing in underserved areas, with existing health inequities, resulted in poorer outcomes in patients with COVID-19, loss of opportunity to mitigate infection transmission, and increased health inequities. These challenges may be partially alleviated by a national system for tracking licensing and credentialling so that HCPs are more easily relocated to areas with high needs.

### Healthcare epidemiologists and infection preventionists role in pandemic preparedness and response

Grounded in specialized training and background, healthcare epidemiologists and infection preventionists have central roles in pandemic preparedness and response.^14^ Healthcare epidemiologists serve as content experts within an overall incident management structure and as leaders of infection prevention and control-related activities. Throughout various phases of the COVID-19 pandemic, the responsibilities of healthcare epidemiologists and infection preventionists have varied in response to evolving community-level transmission and progression. These responsibilities include infection prevention-oriented activities to prevent transmission of SARS-CoV-2 to patients, HCP, learners, and visitors as well as addressing concerns for the spread of other healthcare-associated infections.^15^ Additionally, many healthcare epidemiologists and infection preventionists have taken on expanded roles in community outreach and education. This includes providing expertise to non-healthcare entities on infection control and prevention and COVID-19 vaccination, and engaging with media for dissemination of scientific information and addressing public questions. The capacity to take on expanded roles during a novel pandemic or infectious disease emergency can be limited by institutional support, including allocated time and financial resources. Forthcoming work from SHEA will describe the development, evaluation, and implementation of strategies to support resiliency and sustainability of the Infection Prevention workforce.

## Recommendations to develop the healthcare workforce in preparation for future pandemics (Table 2)

1. Expand the functionality of the existing healthcare workforce, with cross-training of healthcare personnel to adapt to changing needs during a pandemic.
2. Provide tailored education to frontline HCP on infection prevention and specific roles and responsibilities during infectious disease outbreaks with routine ongoing evaluation of competency.
3. Support research on best practices for the implementation and assessment of telemedicine and the expansion of the workforce.
4. Create an evidence-informed framework for providing telemedicine both for diagnosis and treatment of potential pandemic agents and for maintaining routine patient care during a pandemic or crisis.
5. Support collaborations across scientific communities and health systems, to better understand and respond to threats to the healthcare workforce and infrastructure resilience in various settings.
6. Strengthen relationships and collaboration between communities, state and local public health departments, and the healthcare settings they serve.
7. Support expansion of the local healthcare workforce to assist with pandemic preparedness and response activities, including strengthening ties with public healthcare personnel. These partnerships should include diversity, equity, and inclusion in the development of the healthcare workforce.
8. Recognize the expertise of healthcare epidemiologists and infection preventionists and provide dedicated time and resources to support their activities and roles in pandemic preparedness and response across healthcare facilities, including expansion of capacity, when needed.
